# Influence of microgravity on spontaneous calcium activity of primary hippocampal neurons grown in microfluidic chips

**DOI:** 10.1038/s41526-024-00355-x

**Published:** 2024-02-06

**Authors:** Pierre-Ewen Lecoq, Chloé Dupuis, Xavier Mousset, Xavier Benoit-Gonnin, Jean-Michel Peyrin, Jean-Luc Aider

**Affiliations:** 1 PMMH, ESPCI Paris - PSL, Paris, 75005 France; 2 Neurosciences Paris Seine IBPS, UMR8246, Inserm U1130, Sorbonne University, 4 Place Jussieu, Paris, 75005 France

**Keywords:** Neuroscience, Biomedical engineering

## Abstract

The influence of variations of gravity, either hypergravity or microgravity, on the brain of astronauts is a major concern for long journeys in space, to the Moon or to Mars, or simply long-duration missions on the ISS (International Space Station). Monitoring brain activity, before and after ISS missions already demonstrated important and long term effects on the brains of astronauts. In this study, we focus on the influence of gravity variations at the cellular level on primary hippocampal neurons. A dedicated setup has been designed and built to perform live calcium imaging during parabolic flights. During a CNES (Centre National d’Etudes Spatiales) parabolic flight campaign, we were able to observe and monitor the calcium activity of 2D networks of neurons inside microfluidic devices during gravity changes over different parabolas. Our preliminary results clearly indicate a modification of the calcium activity associated to variations of gravity.

## Introduction

Knowing how space flight and associated stress stimuli i.e. hyper- or microgravity, as well as radiation, impact the development of biological organisms is a major concern regarding the future of space exploration^1–4^. From crops to astronauts, it is now essential to understand how the particular environmental conditions of space flight can affect the health of cells and tissues. Upon their return from the ISS, astronauts showed pathological symptoms linked to their prolonged stay in microgravity such as bone density loss, muscle atrophy, cardiovascular and hemodynamic changes, metabolic, endocrine and sleep disturbances^5^. The main physiological effect identified and linked to microgravity that impacts most organs is the blood redistribution across the whole organism. Indeed, the blood pressure induced by the heart no longer needs to overcome the gravity, the vascular system needs less energy to oxygenate primary organs, resulting in less global pressure and making the whole blood circulation more efficient. Aside from this, many other physiological functions can be affected by space-flight conditions. The Twins study lead by NASA (one remained on earth while the other stayed 6 months in the ISS) revealed various effects and risks like mitochondrial dysfunction, immunological stress, vascular changes and cognitive performance decline, as well as alterations in telomere length^4^.

From a neurological point of view, medical studies on astronauts also showed that space flights have effects on the brain both on the short term (motion sickness, vestibular dysfunction and Spaceflight-Associated Neuro-ocular Syndrome) and on the long term (increased risk of brain malignancies, premature aging, accelerated neurodegeneration)^6,7^. In the same manner that microgravity modifies blood pressure, it also causes vestibular and ocular structural dysfunction as the fluid shift increases the intracranial and intraocular pressure leading to SANS (Spaceflight-Associated Neuro-ocular Syndrome)^8,9^. Extensive studies of astronauts alterations of brain structure has recently been substantiated by fMRI analysis of astronauts evidencing subtle yet significant modification of brain connectome^10^. These phenomena are either linked to microgravity itself, via mechanical unloading, or to the intracranial fluid redistribution, or to space radiation. One way or another, understanding the mechanisms underlying the appearance of those symptoms remains an important health concern.

If the physiological studies are important to diagnose the influence of gravity variations on the functioning of various organs, it is also very important to study the possible impacts at the cellular level. Studying cells in microgravity remains a challenge. Usually, long-term studies are needed, which implies cell cultures in the ISS^11,12^. Working with rockets, drop towers or parabolic flights require specific setups and readouts that could show variations during the short experimental times, ranging from a few seconds (drop towers, parabolic flights) to a few minutes (rockets)^13^. From this point of view, neurons^14^ or cardiomyocytes^1^ are very good candidates because of their relatively fast electrical and/or mechanical activities. For these reasons, we chose to study the activity of a 2D network of neurons grown in a microfluidic chip.

To this end, we designed an experimental setup to record live calcium transients of living primary hippocampal neurons, grown inside microfluidic chips, during hyper- and microgravity phases of parabolic flights. To adapt to these particular experimental conditions and constraints, a setup previously used to study self-propulsion of nanorods^15^ has been adapted to the constraints of cell culture and fluorescence microscopy. An inverted fluorescence microscope equipped with a miniaturized cell culture stage incubator and fitted with a fast CMOS camera was packaged in a closed and secure box all remotely controlled via a control rack.

Microfluidic chips were chosen as culture devices for the primary neurons because they show several advantages (for review see^16^). First, they recently emerged as a powerful tool allowing spatial control of neuronal connectivity together with exquisite control of fluidic and chemical micro-environment of the cells. Second, microfluidic chips cell culture platforms are perfectly suited for parabolic flight campaigns as they allow to limit redistribution of fluids and shear stress during the different phases of the flights in order to preferentially record the neuronal activity affected by changes in gravity. Finally, calcium imaging was chosen as readout because of the fast variation in signal due to adaptation to external stimuli. Indeed, during a parabola, the duration of each acceleration phases are very short: 22 s for 0 g and 30 s for 1.8 g. It is a strong limitation to the biological responses that can be monitored. Only fast biological processes, such as ion flux exchanges, can be affected and induce a modification of activity in such a short time. Remarkably, calcium activity of neurons varies quickly, on a few seconds span, with typical frequencies of the order a few Hertz^17,18^. For this reason, one can expect a modification of their firing rates that could be monitored during each gravity phase^19^.

In the following, we will first present the microfluidic chip used to grow an active network of neurons. We will then explain how murine primary hippocampal neurons were obtained and cultivated. The principle of calcium imaging technique will then be briefly presented together with typical activity recorded in laboratory conditions. Then the experimental setup developed to monitor calcium activity during a parabolic flight (164th CNES parabolic flights campaign) will be detailed. Finally, the variations of activity of the 2D networks of neurons will be discussed.

## Methods

### Microfluidic chip for 2D neuronal network

**Fig. 1:**
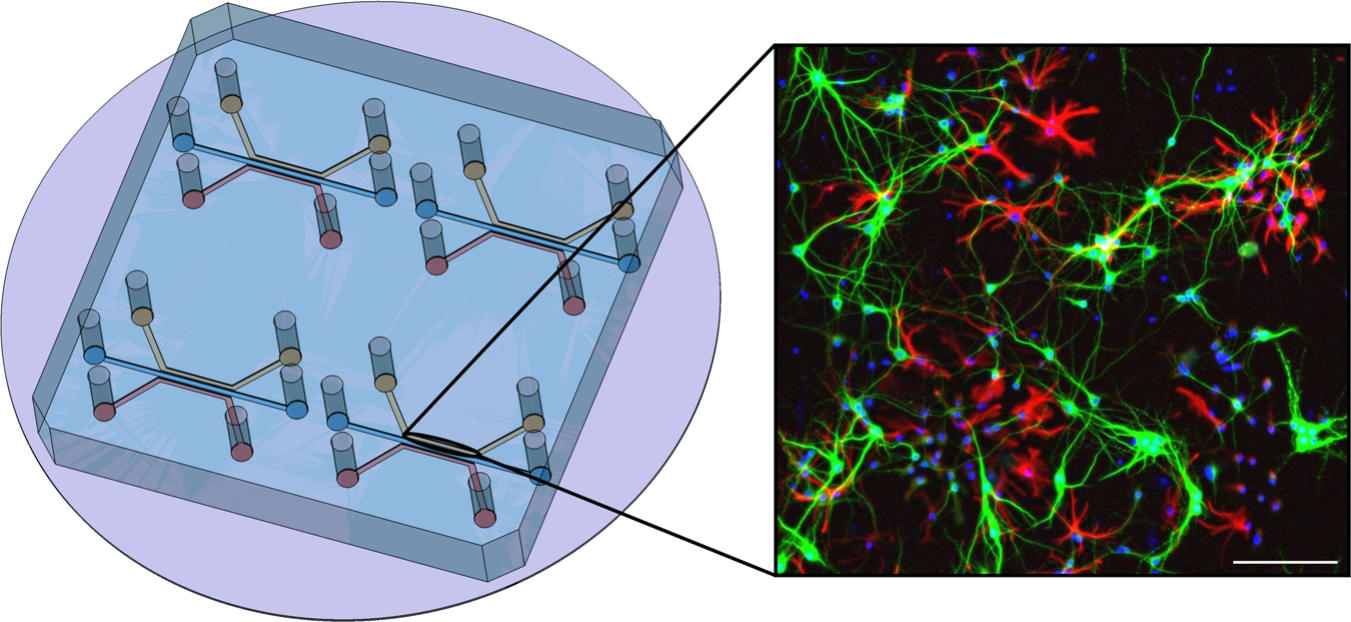
CAD(Computer Aided Design) view of the microfluidic chip used to grow the neuronal networks. The height of the chambers is 50 μm, thus confining the cells in a monolayer. Each inlet is a 50 μL reservoir and the chambers have the following dimensions: 5 mm (length) × 1 mm (width) × 50 μm (height). An immunostaining of a 10-days-old hippocampal neuronal network is also shown. The following dye and primary antibodies were used: DAPI (nucleus) in blue, MAP2 (Microtubule-associated protein 2, showing neurons) in green and GFAP (Glial Fibrillary Acidic Protein, showing astrocytes) in red. Scale bar is 150 μm.

To study the activity of neurons, the first step consisted in designing a proper microfluidic chip allowing for fluid confinement as well as growth of primary cells leading to the creation of a complex 2D network of interconnected and active neurons. A microfluidic chip, similar to the one developed in^20^, containing two independent cell culture chambers, separated by an array of “axonal diodes”, was used. The diodes are asymmetric micro-channels imposing unidirectional axon connectivity with 97% selectivity (measured in^20^) supporting the construction of a complex, oriented neuronal network. Nevertheless, as first proof of concept in the present experiment, only one chamber seeded with primary hippocampal neurons was used (Fig. 1).

### Microfluidic chip fabrication

To fabricate the microfluidic chips suitable for the calcium imaging experiment in the parabolic flight, standard photolithography process was used, based on previous work from Lassus et al. 2018^21^. Briefly, microfluidic silicon wafers were generated trough spin coating of photosensitive resist SU8-2002. They were baked at 65 °C for 30 s and at 95 °C for 2 min. They were then exposed to UV light through a mask for photopolymerization of the resist. The resulting silicon wafers encode 4 microfluidic chips composed of 2 cell culture macro chambers, each of the chambers being connected to an inlet and outlet reservoir. Silicon wafers (or epoxy replicated molds) were used to produce the cell culture PDMS chips. A mixture of Poly Dimethyl Siloxane (PDMS) elastomer and curing agent with a 9:1 ratio was degassed under vacuum to remove air bubbles. The PDMS mix was then cast onto the molds and baked for 4 h at 70 °C. PDMS microfluidic chips were pealed off the wafers, wells for macro chambers were punched manually and the surface of the PDMS was cleaned with tape. PDMS pads were then bonded to glass slides, previously cleaned with iso-propanol as well as by using a plasma cleaner (100% power, 0.6 mBar in O_2_, Diener Electronic). One inlet per chamber was immediately filled with distilled water maintaining the hydrophilicity of both glass and PDMS acquired during the plasma treatment. Finally, the microfluidic chips were sterilized under UV for 30 min. Prior to cell seeding, they were coated with poly-D-lysine (PDL-10 μg/mL diluted in PBS, Sigma3registered) overnight at 37 °C and then with Laminin (2,5 μg/mL diluted in PBS, Sigma®) for 2 h at 37 °C, allowing cells to adhere to the glass slide. Laminin is removed before seeding.

### Cell culture of primary hippocampal neurons

Animal care was conducted in accordance with standard ethical guidelines (U.S. National Institutes of Health publication no. 85–24, revised 1985, and European Committee Guidelines on the Care and Use of Laboratory Animals) and the local, IBPS and UPMC, ethics committee approved the experiments (in agreement with the standard ethical guidelines of the CNRS “Formation á l’Expérimentation Animale” and were approved by the “C2EA -05 Comité d’éthique en experimentation animale Charles Darwin”). Hippocampal neuronal cultures were generated from E18 SWISS mouse embryos by building upon standard cell-culture techniques, as follows. SWISS pregnant mice were purchased from Janvier (Le Genest Saint Isle, France). Animal care was conducted in accordance with standard ethical guidelines. The cell culture preparation was done in the Neurocentre Magendie laboratory in Bordeaux. Hippocampi were micro-dissected from E18 embryos with all steps performed in cold Gey’s Balanced Saline Solution (Sigma G9779). Dissected structures were digested with papaïn (50 U/mL, Sigma 76220) in DMEM (Thermofisher 31966 021). After papaïn inactivation with FBS (100 U/mL, GE Healthcare), structures were mechanically dissociated with a pipette (plastic tip 1 mm diameter) in presence of DNAse type IV (20 U/mL, Sigma D5025). Cells viability was determined by Trypan Blue exclusion assay. The resulting cells were centrifuged 7 min at 800 rpm and the pellet was resuspended in the culture medium containing DMEM, 10% FBS, 2% B27, 0.5 mM Glutamate and 1% Penicillin/Streptomycin (Gibco®) at a concentration of 20 million cells/mL. Cells were seeded in macrochambers and allowed to adhere to the glass surface for 5 to 10 min before filling the rest of the chambers with culture medium. All cell cultures were kept in an incubator at 37 °C and 5% CO2. Typical cultures contain two thirds of neurons and one third of astrocytes which are co-cultured.

### Immunofluorescence staining

To evaluate the integrity of the primary hippocampal neurons inside our microfluidic chips, some chips were fixed in PBS containing 4% paraformaldehyde (PFA, Electron Microscopy Science 15714S) and 4% sucrose (Sigma S9378) for 8 min at room temperature and rinsed twice in PBS for 5 min. Then, they were permeabilized for 30 min at room temperature with 0.2% Triton (Sigma X100) and 1% bovine serum albumin (BSA, Thermofisher 15561020) in PBS. After removing the permeabilizing solution, primary antibodies were added in PBS with 1% BSA and the samples incubated at 4 °C overnight. The samples were rinsed for 5 min with 1% BSA in PBS and further incubated with the corresponding secondary antibodies for 2 h in PBS with 1% BSA at room temperature. Finally, the chips were rinsed first with 1% BSA in PBS and last only with PBS. The following primary antibodies and dye were used: DAPI (4’,6-diamidino-2-phénylindole, nuclear DNA staining) (1050, Euromedex, 1/2000), MAP2 (Microtubule-associated protein 2) (M4403, Sigma, 1/1000), *β*3-tubulin (MA1-118, Thermofisher, 1/1000) and GFAP (Glial Fibrillary Acidic Protein) (Z0334, Dako, 1/1000). Species-specific secondary antibodies coupled to Alexa 350, 488, 555 or 633 were used (1/1000, Invitrogen) to visualize bound primary antibodies.

### Calcium imaging

The calcium dye Fluo4-AM was obtained from ThermoFisher Scientific® (ref F14201). It was dissolved in dimethyl sulfoxide (DMSO) in a ratio of 1 μg: 1 μL to obtain a stock solution of 1 mM. This stock solution was frozen in 2 μL aliquots. *Ca*^2+^ free wash buffer was made according to previous protocols and the stock solution of Fluo4-AM was added in order to load the cells with a final concentration of approximately 2 μM. Before the flights or classical on-ground experiments, a chip with good cell survival was chosen, loaded with Fluo 4 AM and incubated at 37 °C and 5% CO_2_ for 25 min. It was then rinsed with *Ca*^2+^ free wash buffer for 5 min at room temperature. Finally, to protect cells from light as well as temperature variations, the chip was transported in a closed box to the plane or to the on-Earth laboratory imaging facility. The equipment is described in the results section below. Recorded activity was purely spontaneous for all measurements. For both parabolic flights and laboratory experiments, the exposure time was 100 ms every 400 ms. To avoid any photobleaching or phototoxicity effects, for each parabola we picked and imaged a different neuronal network from the four independent channels of the microfluidic chip (Fig. 1).

### Image analysis and detection algorithm

Calcium imaging allows for the visualization of neuronal activity within the neuronal network. In general, segmentation of cells inside a field of view and analysis of fluorescent traces were made with Calima software^22^. For the flight experiments, some parts of the video were too noisy to be analyzed due to an unwanted motion of the microfluidic chip. Thus, a dedicated correction algorithm was applied, based on the Lucas–Kanade method using the optical flow functions of the OpenCv library on Python. This part is described in more details in “Methods”.

## Results

### Monitoring calcium activity

To evaluate the influence of gravity variations on a network of neurons during parabolic flights, one needs a readout for every gravity phase which can vary fast enough in a short time period, i.e. typically 10 s or less. Calcium imaging is particularly adapted as it allows the monitoring of fluorescence fluctuations on many neurons of the network, with typical frequencies around a few *Hz*^18^. It also allows for live video recording for later off-line analysis. Nevertheless, running these kind of measurements in the noisy environment of the Airbus Zero-G, mostly due to the vibration induced by thrusters, remains challenging. For this campaign of parabolic flights, we chose to study the activity of differentiated yet scantly connected neuronal networks. Those immature networks were chosen as they allow to monitor single cell calcium transient that are spontaneous yet not synchronized, and occur without any external stimulation.

### Standard activity in the laboratory

**Fig. 2:**
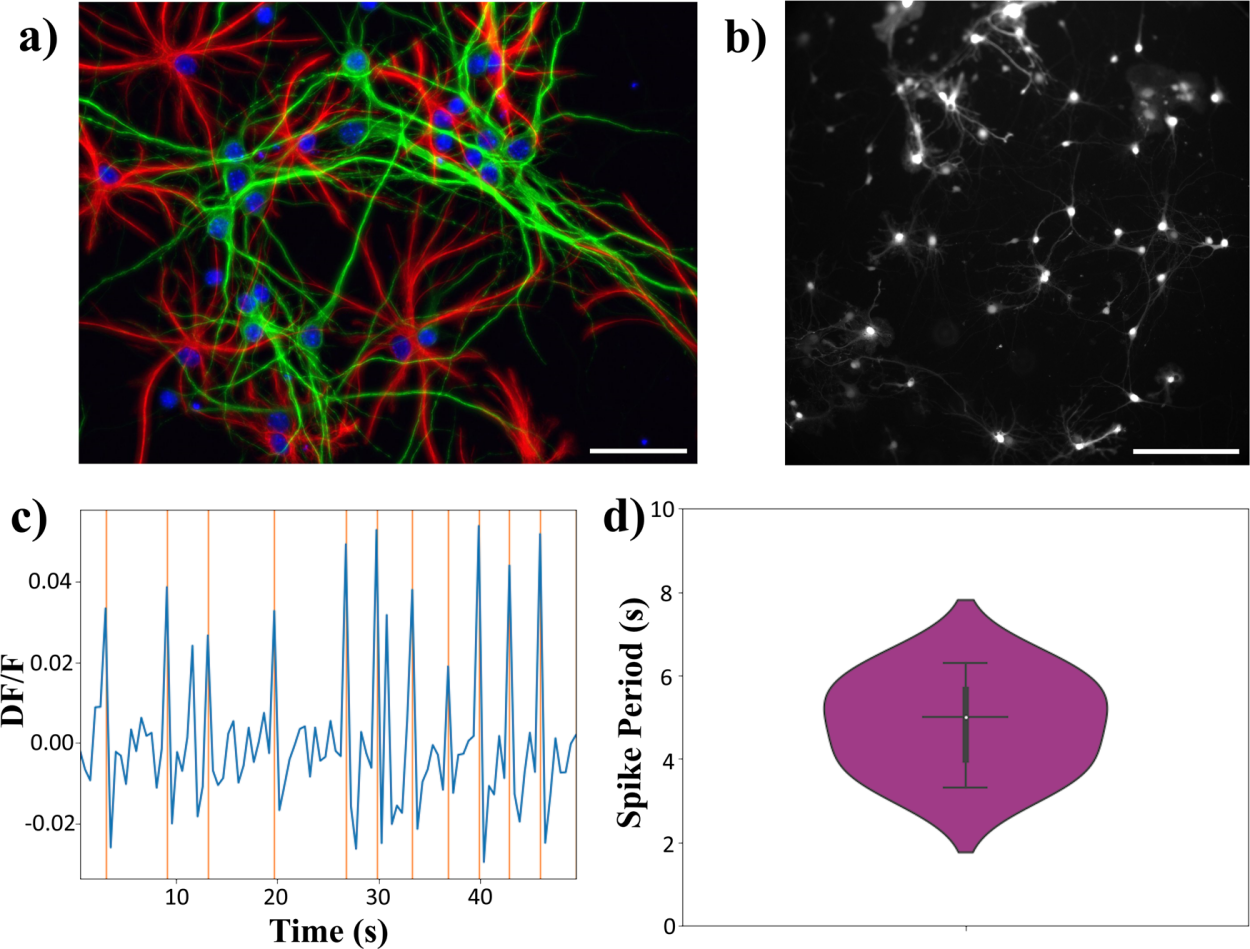
Standard culture and activity in the laboratory. **a** Immunostaining of hippocampus neuronal network with DAPI (blue), MAP2 (green), GFAP (red). Scale bar is 50 μm. **b** Typical view of a 10-days-old hippocampal neuronal network stained with Fluo4 (ThermoFisher Scientific). Scale bar is 100 μm. **c** Example of the normalized calcium activity of one hippocampal neuron, *DF*/*F*. The red lines indicate the peaks detected by the algorithm. **d** Distribution of the oscillation period of calcium activity of immature hippocampal neurons on-ground. The average period is 5 s, white dot represents the median and the black bars the first and third quartiles.

The standard spontaneous activity of 10 days old hippocampus neurons was measured on earth in standard laboratory conditions with the exact same experimental setup that was used during the parabolic flights. Five different chips from different cultures involving different mice were analyzed, with fields of view containing 12, 16, 20, 21 and 27 active neurons. A typical snapshot of a neuronal network is shown in Fig. 2b.

If the network is active (5 of the 6 networks observed showed activity), it is possible to define on the snapshots, specific ROIs (Region of Interest) corresponding to active neuron cells. The time-evolution of the fluorescence signal of a given neuron, i.e. its calcium activity, can then be recorded. In the following, the signal is normalized using $DF/F=\frac{F-{F}_{m}}{{F}_{m}}$ where *F* is the intensity of the chosen ROI and *F*_*m*_ is the mean intensity of the field of view. A typical signal for a given neuron is shown in Fig. 2c. It is then possible to evaluate the period of oscillation of the chosen neuron. The process was repeated for each active neurons identified in the snapshots, leading to a distribution of periods of oscillations observed in the network, as shown in Fig. 2d. One can see a wide distribution of periods of activity, centered around 5 s, and ranging from 2 s to 8 s, meaning we can expect to record a few oscillations during each gravity phase.

The networks were labeled via immunohistochemistry to assess the viability of the cells and the proportion of neurons and astrocytes (Fig. 2a). The viability was evaluated with homemade FIJI routines. Images of fluorescent DNA intercalant labelling (DAPI) were pre-processed and segmented thanks to a Plugin developed by Schmidt and Weigert called StarDist^23,24^. Based on previous works^25–27^, we defined polynucleolated nuclei as alive and mononucleolated, condensed, nuclei with high intensity as dead. The segmentation given by StarDist was further used to decipher the proportion of different cell types of interest. For MAP2+ and GFAP+ cells, as the labeling was somatic, the nuclei segmentation was adapted for the soma by taking each ROI, dilating it before substracting the original one to obtain a ROI with a ring shape. This automated sorting resulted in 65% of living cells with 2 times more neurons than astrocytes.

### Corrections of image distortion

Running microscopy measurements during parabolic flights is challenging because of the many vibrations, leading to possible loss of focus or undesired side motions of the samples. To remove undesired motions (vibrations) and distortions due to local mechanical deformation of the glass wall during the hypergravity phase, a dedicated correction algorithm was developed. The algorithm used is based on the Lucas-Kanade method developed by Bruce D. Lucas and Takeo Kanade in 1981^28^ for computer vision. This method assumes that the displacement between two consecutive images is small and varies little around a given value, but also that the neighboring pixels have a similar speed. It is now commonly used to monitor the motion of objects or even as a visual sensor in closed-loop flow control experiments because of its high-efficiency leading to real-time velocity measurements^29,30^. In the present study we used the optical flow functions from the OpenCv library on Python^31^.

**Fig. 3:**
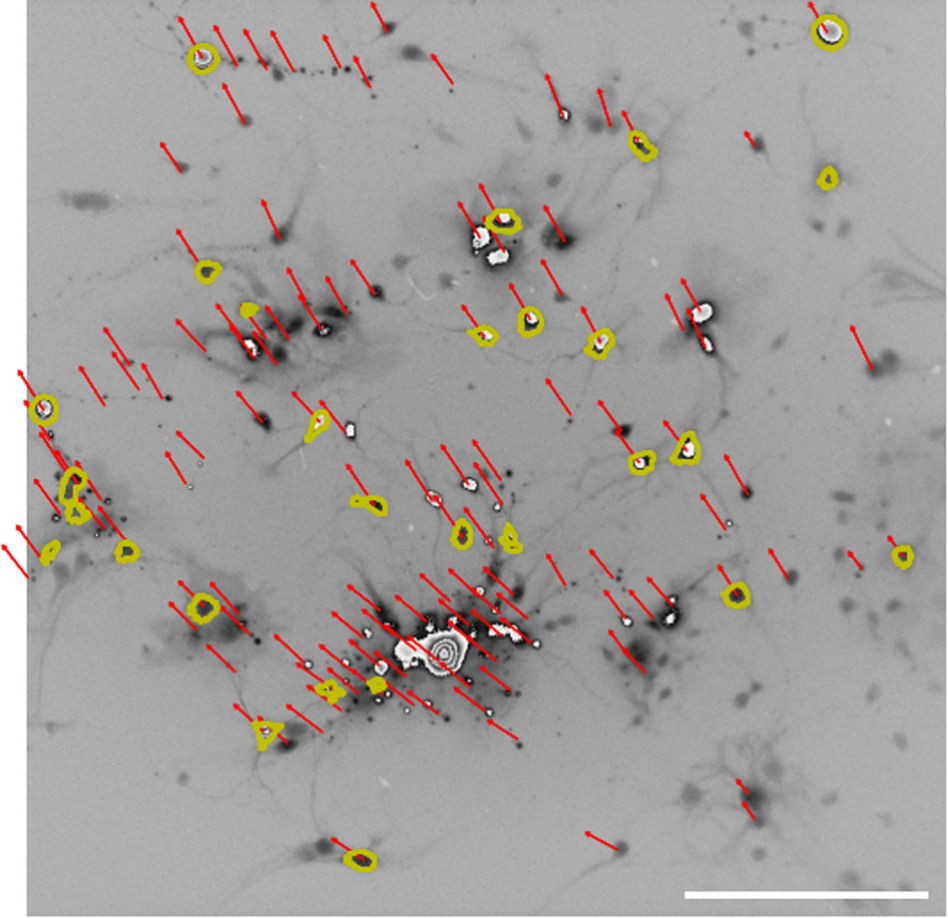
Measurement of the unwanted motions of the sample (of the order of a micron) during parabolic flights. Based on the Lucas-Kanade computer vision method, the displacement of each ROI, corresponding to one neuron, was extracted, leading to corrections before processing the time-series of calcium activity. Scale bar is 100 μm.

The first step was to detect suitable points for the tracking algorithm, in order to follow their movement. In our case, this first step was very efficient thanks to the image resolution and the shape of the objects, leading to a very good measurement of movement. The second step consisted in tracking the objects and deducing the speed associated with the movement. A typical example is shown in Fig. 3 where the undesired motion induced by the vibrations of the experimental setup was measured. Typical displacements measured were close to one micron. This made it possible to stabilize the snapshots by subtracting the measured displacements from the time series.

### Experimental setup adapted to Zero-G flights

To run the calcium imaging experiments in the Airbus Zero-G plane, we modified a dedicated setup composed of three racks, which was previously designed by our team to study acoustic self-propulsion of nanorods^15^. The main new constraints for the setup were related to the manipulation and observation of living cells using fluorescence microscopy in a highly confined environment.

**Fig. 4:**
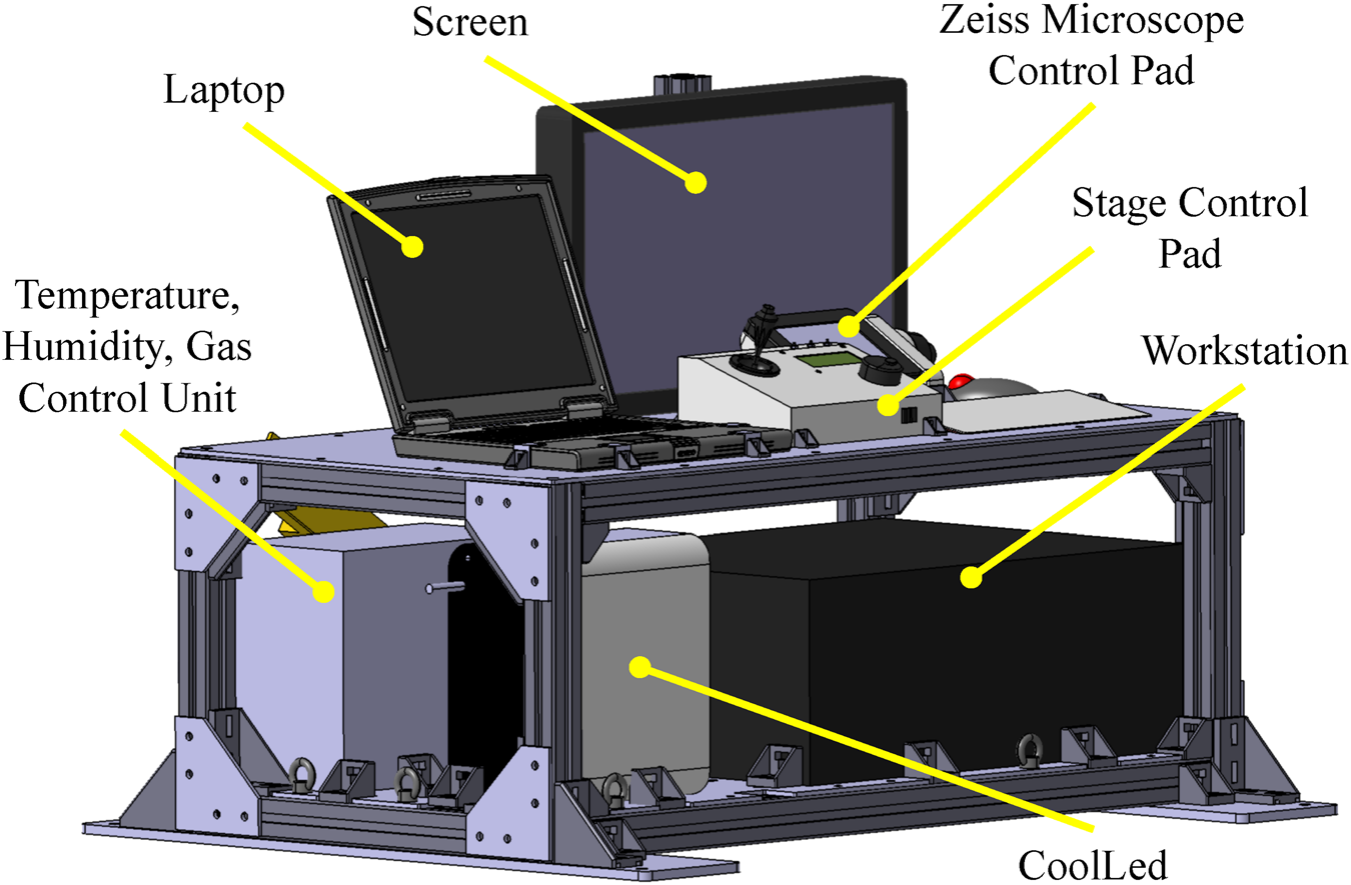
3D view of the monitoring rack (Rack 1). Containing the computers and devices used to control and monitor all the important parameters of the experiment as well as the recording of all data. A fast and highly sensitive camera is connected to the workstation through a USB3 port, allowing the fast acquisition of the snapshots time-series of the visualization of the calcium activity of the 2D neuronal networks.

First, the monitoring rack was conceived to easily control every important parameters of the experiment (acoustic frequency and amplitude, optical illumination, stage controller, etc). It also harbors a workstation connected to a high-speed and highly sensitive camera (PCO Panda Bi) (Fig. 4).

**Fig. 5:**
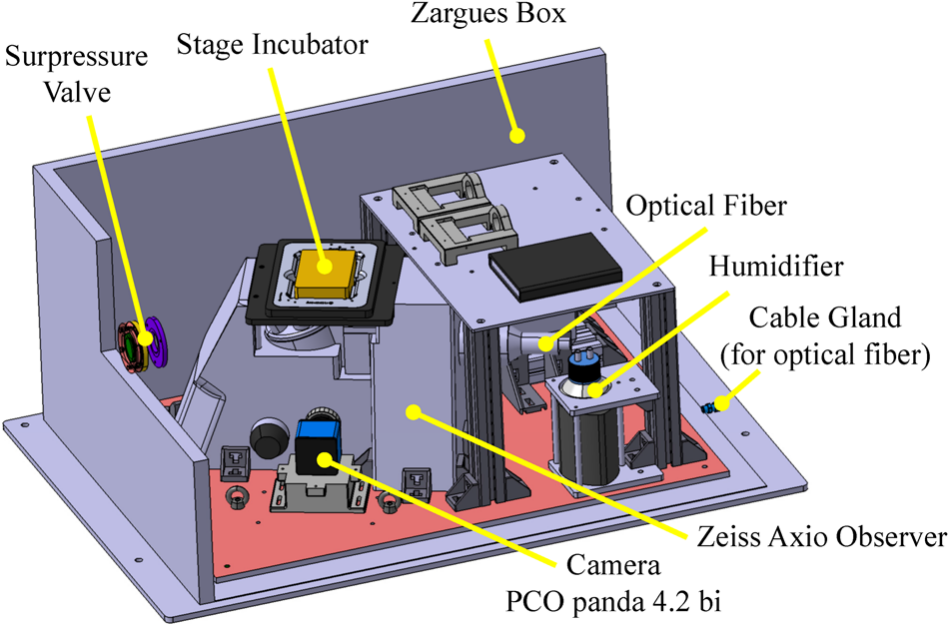
3D view of the experimental box (Rack 2). Inside a sealed Zarges box, we installed a Zeiss inverted microscope with a motorized stage. A stage incubator from TOKAI HIT was used to ensure a well-controlled environment for the cell cultures during the flights. The fluorescence images were recorded using a PCO Panda Bi camera.

**Fig. 6:**
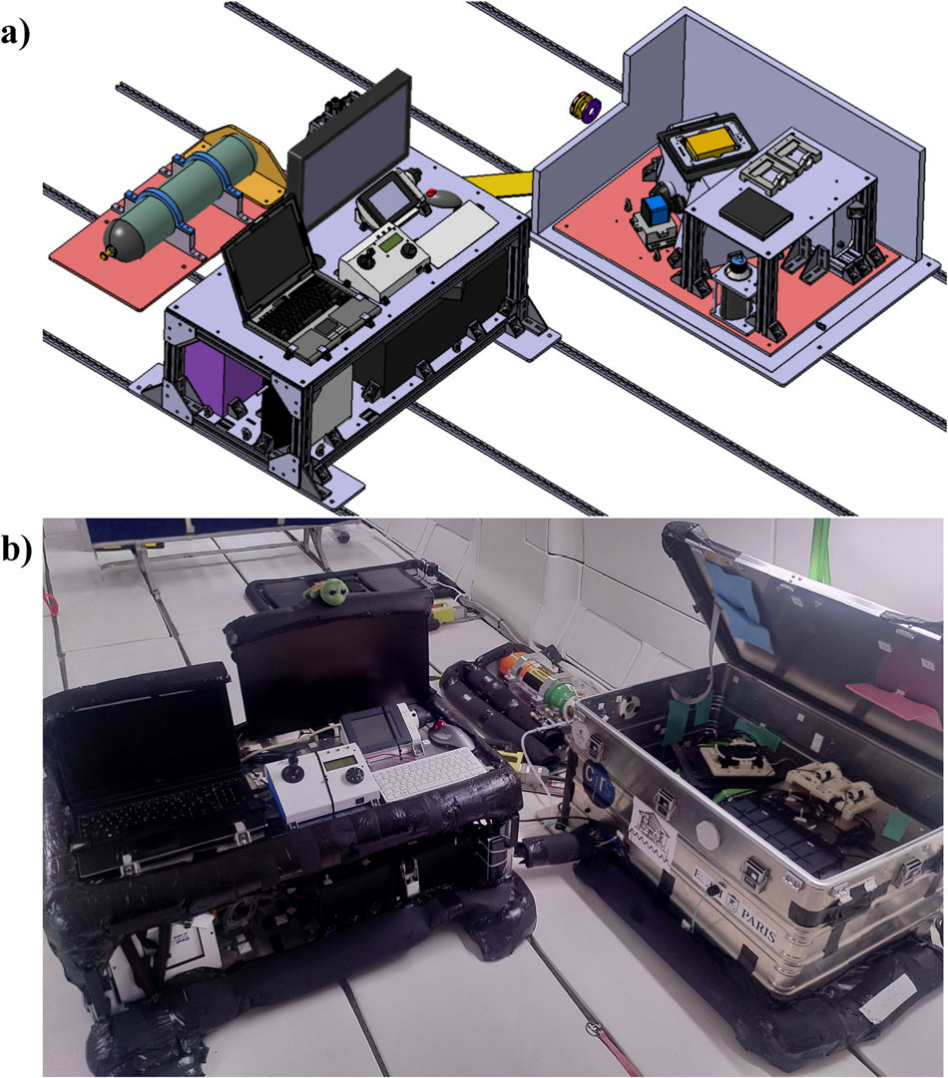
3D overall view of the experiment. **a** 3D view of the full experimental setup (3 racks) used for calcium imaging during the parabolic flights. The racks were fixed on the floor in the Airbus ZeroG as shown on this 3D view. The two racks described above can be operated by two flying operators while the third rack in the back is used to fix the air/*CO*_2_ gas bottle needed for the stage incubator. **b** Picture of the setup installed inside the Airbus A310 ZeroG plane.

Second, the experimental setup was designed to fit into a sealed Zarges box (*Zarges*^*TM*^) to insure a double confinement of liquids (Fig. 5). As previously mentioned, the microfluidic chips were designed to contain a large number of cells in a minimal volume and to be easily sealed with a PDMS layer on top of the inlets/outlets. To be able to record the calcium activity of living neurons during the flight, a stage incubator (TOKAI HIT) was used to control the environmental conditions of the samples (37 °C ± 0.3 °C according to the manufacturer and 5% *CO*_2_ ± 0.1% temperature). In addition, the aircraft is thermally controlled around 18 °C, ensuring partial temperature stability inside the experimental area. The stage incubator was placed on an ASI imaging stage, remotely controlled with a computer installed on Rack 1. Both the stage and stage incubator were fixed on an inverted Zeiss microscope. A CoolLED PE4000 was used for illumination and a PCO Panda Bi camera for image acquisition. A 3D view of the whole experimental set-up as installed in the Airbus A310 ZeroG plane is shown in Fig. 6. The very same setup was used in the laboratory to record the 1 g control experiment of the calcium imaging. The third rack was then used to secure the air/*CO*_2_ mixture bottle on the floor of the plane (Fig. 6). Prior to each flight, the incubator was heated and filled with gas 1 h before take-off.

### Monitoring network calcium activity in Zero-G flights

**Fig. 7:**
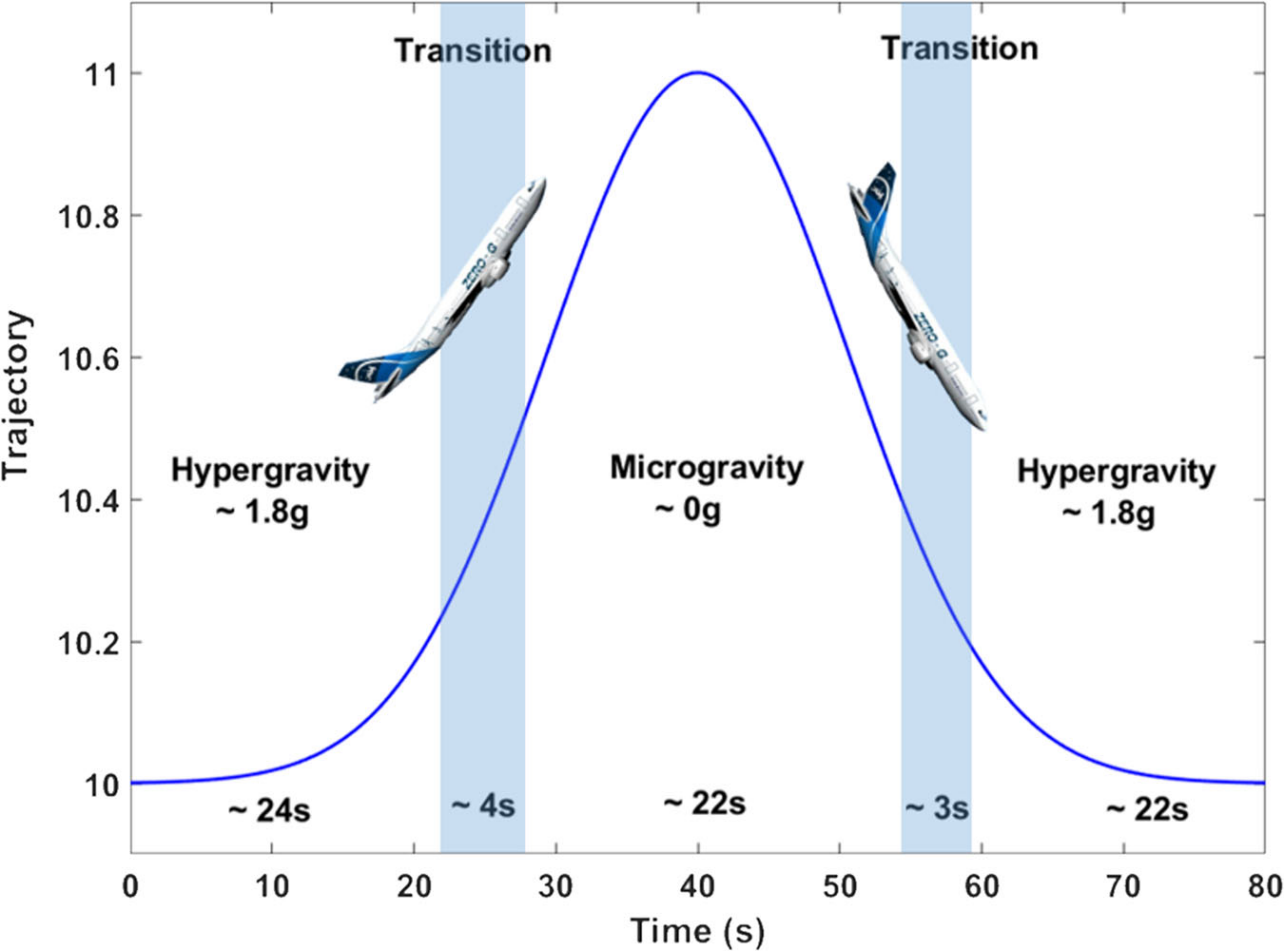
Sketch showing the three phases of gravity during a parabolic flight. It starts with an hypergravity phase, followed by the zero microgravity phase, accurate to 0.1g, before a final hypergravity phase. From Dumy et al.^15^.

During the three flights, we were able to image and record the calcium activity of 29 fields of views from 2 different cultures (i.e different mice), each containing, on average, 6 active neurons (from 2 in one field to 13 at maximum). The recordings were started 10 s before the onset of the parabolas, i.e. before the hypergravity phase (1.8g) and ended 12 s after the offset of the parabola, i.e. the second hypergravity phase (Fig. 7). Every parabola was composed of approximately 30*s* of hypergravity followed by 22*s* of microgravity and finally ending with 30 s of hypergravity. Thus, every recording was approximately 82 s long. Throughout the campaign, we managed to analyze approximately 10*min* of calcium activity of hippocampal primary neurons in hypergravity and more than 10 min in microgravity. Considering that the second hypergravity phase, following the microgravity phase, was never stable enough in acceleration due to adjustments of the plane trajectory. For this reason, we only took into account the first hypergravity phase in the following. In the end, out of all the imaged neurons, around 60% showed a spontaneous activity and half of them reacted to changes in gravity.

**Fig. 8:**
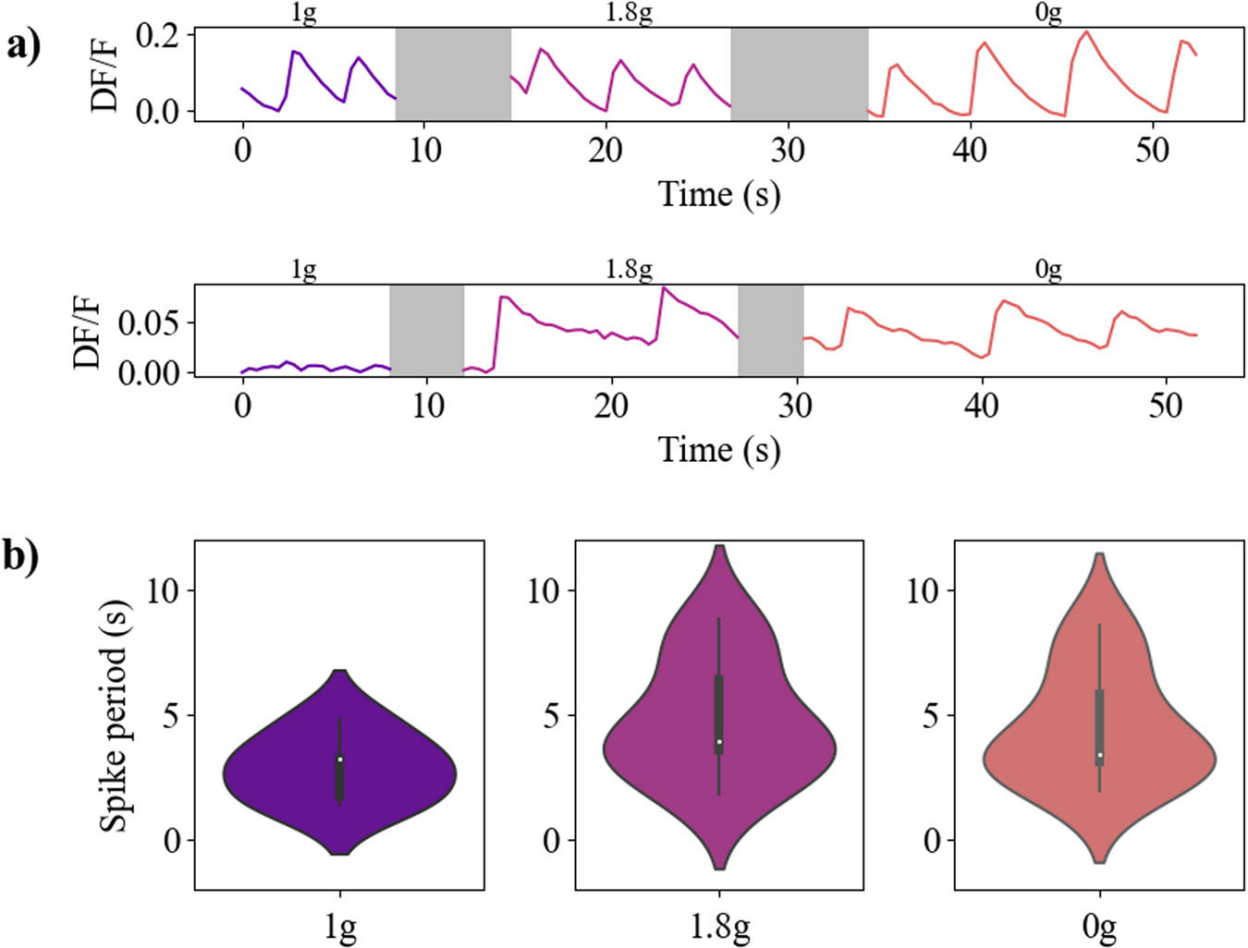
Activity of primary hippocampal neurons during parabolic flights. **a** Typical calcium activity of neurons imaged during a parabola, $DF/F=\frac{F-{F}_{m}}{{F}_{m}}$ where F is the intensity of a chosen region (an active neuron in this case) and *F*_*m*_ the mean intensity value of the field of view. Grey areas correspond to blurred images associated to transition phases in the parabola shown in Fig. 7. The upper graph corresponds to a neuron already active during the 1g phase while the lower graph corresponds to the activity of a neuron triggered by the 1.8 g phase. **b** Violin plots of hippocampal neuronal networks activity for each of the three phases of gravity.

Interestingly, many neurons didn’t show activity at first, in the 1g phase, but started being active during the hypergravity phase and then preserved activity for the rest of the parabola duration. Two typical time-series of calcium activity variations during the three phases of gravity are shown in Fig. 8a. One can qualitatively see the modifications of the activity, both in amplitude and period, for the hyper- and microgravity phases compared to the 1 g phase. Figure 8b shows a more quantitative analysis through violin plots. We can clearly see an increase in the average oscillation period (from 2.9 s, *σ* = 1.54, in 1 g to 4.8 s, *σ* = 2.09, in 1.8 g and 5 s, *σ* = 2.09, in 0 g), associated to a much wider distribution of the period of oscillation.

We also observed a modification of the envelope of the violin distribution. Indeed, a bi-modal distribution of the neuronal activity period arises during both the microgravity and hypergravity phases (Fig. 8b), which can be explained by the activation of neurons with a slower activity. However, it is difficult in the present experiment, due to the constraints of parabolic flights, to evaluate whether this change of activity was triggered by the 1.8 g phase and maintained in the 0 g phase or if it was also a response caused by the 0 g phase itself.

**Fig. 9:**
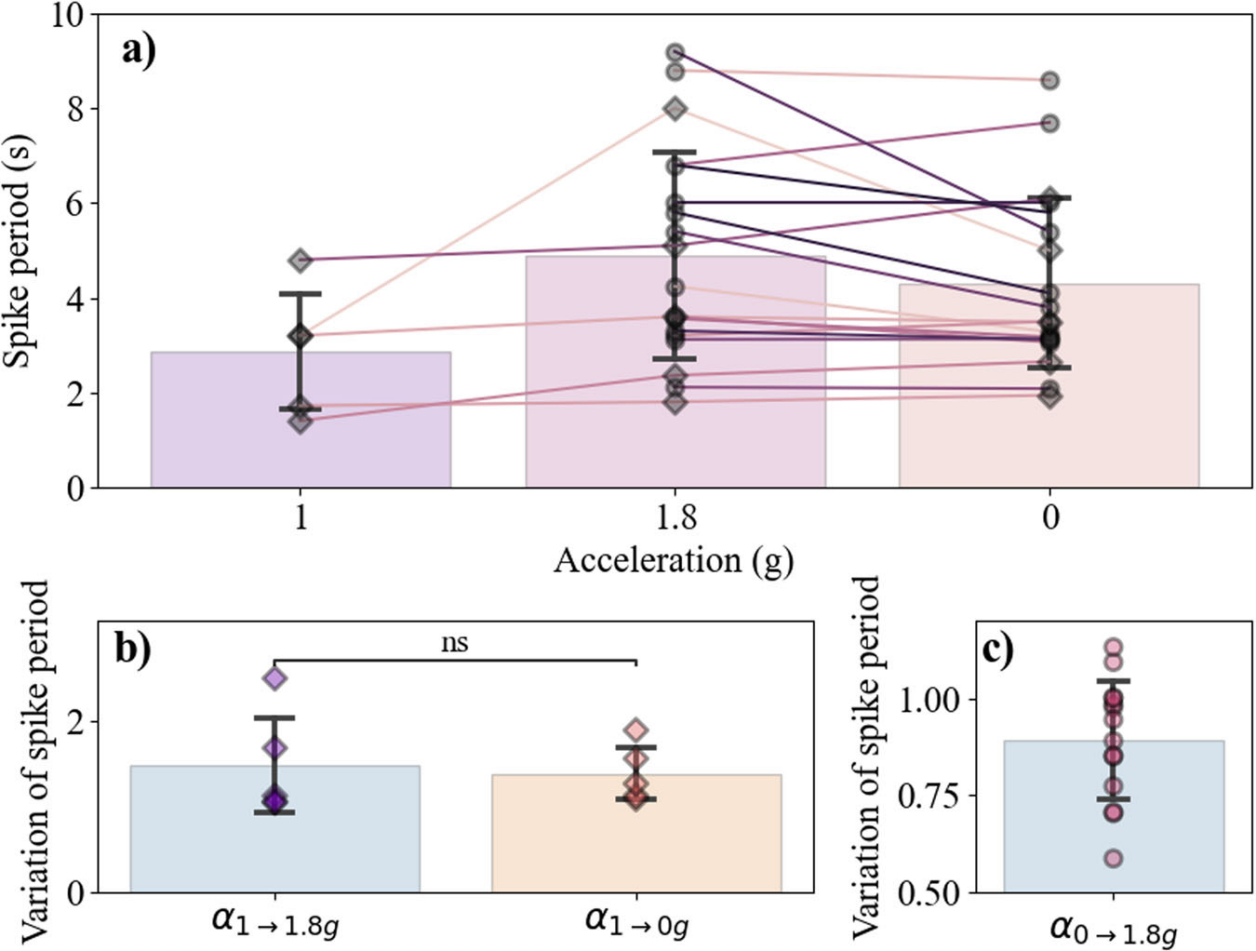
Comparison of activity of primary hippocampal neurons during parabolic flights. **a** Diagram showing the spike period of active neurons computed for each of the three phases of gravity. Each point corresponds to the average spike period for one field of view containing between 2 and 13 neurons. Diamonds represent groups of neurons with activity along the entire parabola, while circles show groups of neurons that begin firing along the 1.8 g phase. The points that are linked are associated to the same group of neurons. Measured spike periods are: 2.9*s*, *σ* = 1.54, in 1g; 4.8*s*, *σ* = 2.09, in 1.8 g; 5 s, *σ* = 2.09, in 0g. **b** Relative spike period variation of the neurons compared to the 1g phase for those which already presented activity at 1 g. **c** Relative spiking period variation of the neurons between the 1.8 g and 0 g phases, for those which did not present activity at 1 g.

Nevertheless, Fig. 9 shows a more detailed analysis of the activity of the neuronal network. Fig. 9a shows the evolution of the mean spike periods for different sets of neurons during the three phases of gravity. From Fig. 8, we first observe that the spike period seems to increase in the hyper and microgravity compared to the 1 g case. It is confirmed quantitatively in Fig. 9b where we show the ratio of spike activity compared to the 1g phase. The median variations of spike period between 1 g and 1.8 g phases *α*_1→1.8*g*_ and between 1 g and 0g phases *α*_1→0*g*_ are both superior to 1, showing that the global network activity indeed slows down. We also notice that the dispersion of *α*_1→1.8*g*_ is bigger than *α*_1→0*g*_ even though the two distributions are statistically equivalent.

The second important observation was the triggering of the activity of neurons that were not previously active in 1 g. In Fig. 9a, we can see nearly four times more active networks in the 1.8 g and 0 g phases than in the 1 g phase. Indeed, 5 neural networks were active from the start of phase 1 g then throughout the parabola, compared to 14 which did not show activity at 1 g but were activated during the hypergravity phase. For all these networks, once neuronal activity was triggered in the 1.8 g phase, the neurons remained active in the 0 g phase. Furthermore, from Fig. 9c, one can also infer that the spike period of these newly activated neurons was, in average, reduced in the 0g phase compared to the 1.8 g phase, as the median of *α*_1.8→0*g*_ is below 1. This means that the networks activated by the hypergravity phases mostly had a faster activity in microgravity. Overall, these results confirm the influence of the gravity variations on the calcium activity of immature hippocampal neuronal networks.

## Discussion

There have been relatively few studies on the effects of weightlessness on the brain, and yet, decades of observations on space-flown animal models and human astronauts have confirmed that physiology is profoundly impacted by spaceflight^32–34^. Growing evidences show that prolonged exposure to spaceflight and microgravity environment leads to alteration of brain function and structure, as suggested by recent analyses of astronauts’ brain fMRI^10^. Even at a smaller scale, microgravity is known to have a direct effect on cells, such as the modification of their cytoskeleton or changes in their physical properties^3,35^. While neuronal connectivity and brain activity are highly dynamic and intermingled processes, this calls for the detailed study of the impact of microgravity and/or space conditions on the intimate functions of neuron networks.

Probing alteration of brain connectome and neuronal networks functions under microgravity conditions remains a very challenging task. This, indeed, relies on the recording of fast and transient signals generated by brain networks typically recorded trough EEG^36,37^ at the whole brain level, or through electrophysiological or calcium imaging studies at the cellular (neuronal) level. While many studies have been developed to monitor the differences in the behavior of mammalian cells maintained in cell culture under simulated or real microgravity environment^11,14,38,39^, most of those have been conducted without live imaging at the cell level.

In order to tackle these issues, our goal was to setup and validate an integrated and automated live cell imaging platform for recording fast calcium transients in living neuronal networks during parabolic flight campaigns. This encompassed the adaptation to Zero-G airplane confinement and security prerequisite, like fluid management, gas leaking security, and vibrations. It required the engineering of a conventional setup composed of an inverted automatic microscope setup, fitted with a cell culture incubator, fast LED illumination and CMOS camera.

One issue was to choose the proper cells to grow a neuronal network, taking into account the constraints of zero-g flights. In this very specific environment, while manipulating human derived neurons would be of great interest, handling the cells during a parabolic flight remains an issue for security reasons. To benchmark our experimental platforms and still obtain relevant results, we therefore chose to handle mouse-derived neurons.

A microfluidic cell culture environment presented several advantages compared to conventional ones during parabolic flights. Indeed, beside its high versatility and fast prototyping capacity allowing to tailor cell culture chambers for specific experiments, microfluidic chips allowed excellent control of the fluidic environment during parabolas. Sealing the microchips inlet and outlets allowed to avoid back and forth cell medium movement and therefore avoided any risk of fluid spillover and allowed mitigation of fluid shear stress during tilting of the plane. The fortuitous occurrence of micro-bubbles in the microfluidic chips was similarly mitigated by the difference in aspect ratio of cell culture chambers and inlet/outlet reservoirs, bubble being trapped in high aspect ratio inlets or outlets. Besides, there is interest in building organ on chip to probe complex physiological or pathological scenarios in space, which demonstrates the clear advantages of using microfluidic cell culture environment in zeroG or gravity variation conditions.

Our calcium imaging protocol was based on a non-ratio metric assessment of intracellular calcium variation through the use of Fluo4-AM, a cell-permeable organic chemical probe widely used in soil experiments as a reliable way to assess neuronal activity. While one of the drawbacks of fluorescent chemical probes is progressive bleaching and phototoxicity, to avoid any bias in our recordings we implemented a careful optimization of acquisition parameters (limited exposure time, high sensitivity camera) and a strategy of cellular sampling in microfluidic devices to record neuronal areas not previously illuminated in previous parabolas. The last (unstable) 1 g phase of parabolas was used to select a new region of interest. Should longer recording times be required (multiple parabolas or long-term zero-G environments such as the ISS or microsatellite environments), genetically encoded *Ca*^2+^ sensors such as GCaMPs with high dynamic range, faster signal integration and no photobleaching would be more suitable^40^. This option will be evaluated during future parabolic flights campaigns.

Furthermore, while we matched media and environmental conditions for ground and flight samples as closely as possible, we note additional variables. Different activity on the ground compared to 1 g in the plane, may be due to take-off accelerations, as well as temporary changes in experimental conditions (heating and *CO*_2_ intakes are turned off for safety reasons during take-off which takes around 30 min), although the influence of the temperature during the flight was controlled by the stage top incubator when acquiring calcium imaging data. These are limitations related to the particular conditions of parabolic flights that can not be avoided in the current setup.

Overall, using the aforementioned newly developed platform and this experimental protocol, we succeeded in recording the spontaneous activity of neuronal networks derived from rodent primary neurons, grown in microfluidic chips, during parabolic flights. We observed important variations of neuronal oscillations that encompassed two distinct phenomena. First, a slowdown of the spontaneous activity frequency due to the variation of gravity, as the period between spikes increased in 1.8 g and 0 g conditions compared to 1 g. Second, we observed significant changes in the percentage of active neurons. Indeed, the transition from 1 g to 1.8 g led to the activation of silent, non oscillating, neurons that remained active during the rest of the parabola. While this substantiates the idea that changes in gravity modify neuronal oscillatory behavior, our results raise several questions.

First, we noticed that both neuronal firing probability and frequency of oscillation were similarly affected by 1.8 g and 0 g conditions. This is in line with previous observations that showed that while 0 g led to membrane hyperpolarizarion and 1.8*g* led to depolarization, intracellular calcium levels of immortalized neuronal cell lines rose as well under hyper as under microgravity^41^. More recently, a study performed on human iPSC derived neurons interfaced with MEA (Micro-Electrodes Array) recording platforms evidenced slight increases in network’s bursting frequency during both 0 and 1.8 g phases in a drop tower experimental paradigm^14^. This raises the possibility that a memory or hysteresis effect occurs at the cell membrane level that lasts during the following gravity phases.

Second, our results show that 0 g (and 1.8 g) conditions lead to a decrease in neuronal firing rates. While this may seem contradictory with results showing that intracellular calcium level increases in SY5Y immortalized cell lines and iPSC human neurons, our experimental platform significantly differs from the previously described ones. Indeed, we recorded the single cell activity of relatively young, immature, primary hippocampal neurons. At that developmental stage, hippocampal neurons behave as self-sustained oscillators^42^ in a non-synchronous manner regarding the rest of the network. On the contrary, at later developmental stages, massive networks show synchronous activity which may arise through a percolation process and the setting up of both feedforward and feedback activity loops between excitatory and inhibitory neurons contained in the culture^43,44^. Importantly, both our study and the one performed in drop tower show that changes in gravity significantly activate silent or very slow firing rate neurons. This clearly suggests that gravity induced neuronal activity changes may differ depending on the neuronal subtypes. On top of this, astrocytes, which are known to buffer neuronal activity, have been shown to be impacted by hypergravity^45^. Therefore, a formal comparison between the spontaneous and/or evoked activity of both immature and mature neuronal networks, together with a method allowing to correlate calcium signals with neuronal subtype identities, would be required to clearly decipher the consequences of acute gravity variation on brain networks activity.

In their review, Florian Kohn and Ramona Ritzmann^46^ discuss a first model of neuronal short-term adaptation to changes in gravity which seems adapted to the timescale of parabolic flights. This takes into account the findings on the different levels of organization predicting opposing results on neuronal firing pattern depending on network connectivity. Acknowledging that microgravity conditions modify neuronal firing rate and that long term changes in gravity lead to modification of synaptic connectivity^47^, it is therefore tempting to speculate that complex cellular processes, such as synaptic homeostatic plasticity adaptations (a cellular and molecular program used to reset global network activity under chronic activity changes^48^), may be at play during long term gravity changes. Challenging this hypothesis would require longer recording times, over several parabolas. It would allow to study how neuronal networks integrate and self-adapt to such transient and periodic changes at the whole population level. One improvement for future parabolic flights campaigns could be to observe the activity of more mature cells in order to be able to analyze the influence of gravity variations on network dynamics and not only on isolated neurons.

As humans will spend more time in space, we must better understand the effects of spaceflight on human physiology at the cellular level. In the present study, we demonstrated that live-imaging of primary neurons can be achieved in parabolic flights, leading to a clear demonstration of the modifications of neuronal activity by changes in gravity. Further investigations into neuronal response to spaceflight are necessary to confirm these results and elucidate the mechanisms of response to gravity modifications. Improvements can be achieved by using three-dimensional, tissue-like structures, such as cerebral organoids, which would provide a more physiologically accurate model and allow the study of interactions between multiple cell types^49^. At longer time scales, astronauts do not suffer from dramatic modification of neuronal activities even if they show cognitive impairment and fine motor control loss. Certainly, even though gravity variation induce transient changes in rhythmic activity, several modifications of cells homeostasis occur at whole network levels to reset normal global activity through molecular adaptations.

## References

[1] Acharya A (2019). Parabolic, flight-induced, acute hypergravity and microgravity effects on the beating rate of human cardiomyocytes. Cells.

[2] Vernós, I., González-Jurado, J., Calleja, M. & Marco, R. Microgravity effects on the oogenesis and development of embryos of drosophila melanogaster laid in the spaceshuttle during the biorack experiment (esa) 10.1387/ijdb.052077sc. 2518159

[3] Crawford-Young SJ (2006). Effects of microgravity on cell cytoskeleton and embryogenesis. Int. J. Dev. Biol..

[4] Garrett-Bakelman FE (2019). The nasa twins study: a multidimensional analysis of a year-long human spaceflight. Science.

[5] Demontis, G. C. et al. Human Pathophysiological Adaptations to the Space Environment. *Front. Physiol*. **8**, https://www.frontiersin.org/articles/10.3389/fphys.2017.00547 (2017). 10.3389/fphys.2017.00547 PMC5539130 28824446

[6] Roy-O’Reilly M, Mulavara A, Williams T (2021). A review of alterations to the brain during spaceflight and the potential relevance to crew in long-duration space exploration. NPJ Microgravity.

[7] Lee JK (2019). Spaceflight-associated brain white matter microstructural changes and intracranial fluid redistribution. JAMA Neurol..

[8] Hupfeld KE, McGregor HR, Reuter-Lorenz PA, Seidler RD (2021). Microgravity effects on the human brain and behavior: dysfunction and adaptive plasticity. Neurosci. Biobehav. Rev..

[9] Gupta, U., Baig, S., Majid, A. & Bell, S. M. The neurology of space flight: how does space flight effect the human nervous system? Life Sciences in Space Research https://www.sciencedirect.com/science/article/pii/S2214552422000694 (2022). 10.1016/j.lssr.2022.09.003 36682819

[10] Jillings S (2023). Prolonged microgravity induces reversible and persistent changes on human cerebral connectivity. Commun. Biol..

[11] Wnorowski A (2019). Effects of spaceflight on human induced pluripotent stem cell-derived cardiomyocyte structure and function. Stem Cell Rep..

[12] Cepeda C (2019). Human neural stem cells flown into space proliferate and generate young neurons. Appl. Sci..

[13] Ferranti F, Del Bianco M, Pacelli C (2020). Advantages and limitations of current microgravity platforms for space biology research. Appl. Sci..

[14] Striebel J (2023). Human neural network activity reacts to gravity changes in vitro. Front. Neurosci..

[15] Dumy G (2020). Acoustic manipulation of dense nanorods in microgravity. Microgravity Sci. Technol..

[16] Habibey R, Rojo Arias JE, Striebel J, Busskamp V (2022). Microfluidics for neuronal cell and circuit engineering. Chem. Rev..

[17] Chen X (2023). Granger causality analysis for calcium transients in neuronal networks, challenges and improvements. Elife.

[18] Eckmann J-P (2007). The physics of living neural networks. Phys. Rep..

[19] Grienberger, C. & Konnerth, A. Imaging calcium in neurons. **73**, 862–885 https://linkinghub.elsevier.com/retrieve/pii/S0896627312001729. 10.1016/j.neuron.2012.02.011 22405199

[20] Peyrin J-M (2011). Axon diodes for the reconstruction of oriented neuronal networks in microfluidic chambers. Lab a Chip.

[21] Lassus B (2018). Glutamatergic and dopaminergic modulation of cortico-striatal circuits probed by dynamic calcium imaging of networks reconstructed in microfluidic chips. Sci. Rep..

[22] Radstake FDW, Raaijmakers EAL, Luttge R, Zinger S, Frimat JP (2019). CALIMA: The semi-automated open-source calcium imaging analyzer. Comput. Methods Programs Biomed..

[23] Schmidt, U., Weigert, M., Broaddus, C. & Myers, G. Cell detection with star-convex polygons, 265–273 (Springer International Publishing, 2018) 10.1007/978-3-030-00934-2_30.

[24] Weigert, M., Schmidt, U., Haase, R., Sugawara, K. & Myers, G. Star-convex polyhedra for 3d object detection and segmentation in microscopy. In *2020 IEEE Winter Conference on Applications of Computer Vision (WACV)* (IEEE, 2020) 10.1109/WACV45572.2020.9093435.

[25] Shiels C, Adams NM, Islam SA, Stephens DA, Freemont PS (2007). Quantitative analysis of cell nucleus organisation. PLOS Comput. Biol..

[26] Ferro, A. et al. Blue intensity matters for cell cycle profiling in fluorescence DAPI-stained images. *Lab. Invest.; J. Tech. Methods Pathol.* **97**, 615–625 (2017). 10.1038/labinvest.2017.13 28263290

[27] Priyanka, R., Arcot, S., Erik, M. & Yang, S. Estimation of three-dimensional chromatin morphology for nuclear classification and characterisation. 10.1038/s41598-021-82985-9 (2021) 10.1038/s41598-021-82985-9 PMC7873284 33564040

[28] Lucas, B. D. & Kanade, T. An iterative image registration technique with an application to stereo vision. In *IJCAI'81: 7th international joint conference on Artificial intelligence*, Vol. 2, 674–679 (1981).

[29] Gautier N, Aider J (2015). Real-time planar flow velocity measurements using an optical flow algorithm implemented on gpu. J. Vis..

[30] Gautier N, Aider J-L (2015). Frequency-lock reactive control of a separated flow enabled by visual sensors. Exp. Fluids.

[31] Bradski, G. The openCV library. *Dr. Dobb’s J: Soft. Tools Pro. Prog*. **25**, 120–123 (2000).

[32] Demertzi A (2016). Cortical reorganization in an astronaut’s brain after long-duration spaceflight. Brain Struct. Funct..

[33] Jillings S (2020). Macro-and microstructural changes in cosmonauts’ brains after long-duration spaceflight. Sci. Adv..

[34] Hong X, Ratri A, Choi S (2021). Effects of spaceflight aboard the international space station on mouse estrous cycle and ovarian gene expression. NPJ Microgravity.

[35] Janmaleki M, Pachenari M, Seyedpour SM, Shahghadami R, Sanati-Nezhad A (2016). Impact of Simulated Microgravity on Cytoskeleton and Viscoelastic Properties of Endothelial Cell. Sci. Rep..

[36] Schneider, S., Brümmer, V., ans Heather Carnahan, A. M., Dubrowski, A. & Strüder, H. K. Increased brain cortical activity during parabolic flights has no influence on a motor tracking task. 10.1007/s00221-007-1187-6. 10.1007/s00221-007-1187-6 17973100

[37] Schneider S (2008). What happens to the brain in weightlessness? a first approach by eeg tomography. NeuroImage.

[38] Yoon N, Na K, Kim HS (2017). Simulated weightlessness affects the expression and activity of neuronal nitric oxide synthase in the rat brain. Oncotarget.

[39] Hammer, A. et al. Retrograde analysis of calcium signaling by campari2 shows cytosolic calcium in chondrocytes is unaffected by parabolic flights. *Biomedicines,* **10** https://www.mdpi.com/2227-9059/10/1/138 (2022). 10.3390/biomedicines10010138 PMC8773224 35052817

[40] Barnett LM, Hughes TE, Drobizhev M (2017). Deciphering the molecular mechanism responsible for GCaMP6m’s Ca2+-dependent change in fluorescence. PloS one.

[41] Kohn, F. High throughput fluorescent screening of membrane potential and intracellular calcium concentration under variable gravity conditions. *Microgravity Sci. Technol*. 10.1007/s12217-012-9331-8.

[42] Penn, Y., Segal, M. & Moses, E. Network synchronization in hippocampal neurons. 10.1073/pnas.1515105113. 10.1073/pnas.1515105113 PMC4812773 26961000

[43] Eckmann, J., Moses, E., Stetter, O., Tlusty, T. & Zbinden, C. Leaders of neuronal cultures in a quorum percolation model. 10.3389/fncom.2010.00132. 10.3389/fncom.2010.00132 PMC2955434 20953239

[44] Breskin, I., Soriano, J., Moses, E. & Tlusty, T. Percolation in living neural networks. 10.1103/PhysRevLett.97.188102. 10.1103/PhysRevLett.97.188102 17155581

[45] Lichterfeld, Y. et al. Hypergravity attenuates reactivity in primary murine astrocytes. Biomedicines **10** https://www.mdpi.com/2227-9059/10/8/1966 (2022). 10.3390/biomedicines10081966 PMC9405820 36009513

[46] Kohn, F. & Ritzmann, R. Gravity and neuronal adaptation, in vitro and in vivo-from neuronal cells up to neuromuscular responses: a first model. *Eur. Biophys. J.* (2018). 10.1007/s00249-017-1233-7 PMC5834568 28656475

[47] Pani, G. et al. Morphological and physiological changes in mature in vitro neuronal networks towards exposure to short-, middle- or long-term simulated microgravity. (2013). 10.1371/journal.pone.0073857 PMC3774774 24066080

[48] Lu, C., Xiling, L., Michelle, T. & Shruti, T. Homeostatic plasticity and excitation-inhibition balance: The good, the bad, and the ugly. *Curr. Opin. Neurobiol*. 10.1016/j.conb.2022.102553. 10.1016/j.conb.2022.102553 PMC9477500 35594578

[49] Wnorowski A, Yang H, Wu JC (2019). Progress, obstacles, and limitations in the use of stem cells in organ-on-a-chip models. Adv. Drug Deliv. Rev..

